# Simulated Macro-Algal Outbreak Triggers a Large-Scale Response on Coral Reefs

**DOI:** 10.1371/journal.pone.0132895

**Published:** 2015-07-14

**Authors:** Justin Q. Welsh, David R. Bellwood

**Affiliations:** 1 MScience Pty Ltd, Perth, WA, Australia; 2 Australian Research Council Centre of Excellence for Coral Reef Studies, and College of Marine and Environmental Sciences, James Cook University, Townsville, Australia; Leibniz Center for Tropical Marine Ecology, GERMANY

## Abstract

Ecosystem degradation has become common throughout the world. On coral reefs, macroalgal outbreaks are one of the most widely documented signs of degradation. This study simulated local-scale degradation on a healthy coral reef to determine how resident taxa, with the potential to reverse algal outbreaks, respond. We utilized a combination of acoustic and video monitoring to quantify changes in the movements and densities, respectively, of coral reef herbivores following a simulated algal outbreak. We found an unprecedented accumulation of functionally important herbivorous taxa in response to algal increases. Herbivore densities increased by 267% where algae were present. The increase in herbivore densities was driven primarily by an accumulation of the browsing taxa *Naso unicornis* and *Kyphosus vaigiensis*, two species which are known to be important in removing macroalgae and which may be capable of reversing algal outbreaks. However, resident individuals at the site of algal increase exhibited no change in their movements. Instead, analysis of the size classes of the responding individuals indicates that large functionally-important non-resident individuals changed their movement patterns to move in and feed on the algae. This suggests that local-scale reef processes may not be sufficient to mitigate the effects of local degradation and highlights the importance of mobile links and cross-scale interactions.

## Introduction

Ecosystem degradation is a common problem faced throughout the world, with changes compromising the complexity and productivity of ecosystems (e.g., [1], [2], [3], [4]). In many systems, the recovery of ecosystem communities and processes relies strongly on ‘mobile links’ [5], [6]. Mobile links are taxa with large-scale movements that act as vectors (cross scale interactions [7]) transferring essential elements of recovery from relatively healthy systems to more degraded ones [8]. In tropical rainforests, fruit bats and bird taxa are good examples of such mobile links. Through their large-scale movements, seeds originating from healthy fruit trees are dispersed in faecal matter over a wide area [9], [10]. This is especially important for ecosystem recovery, as the seeds transported by the bats and birds may be deposited in degraded areas where the mature forest canopy has been removed and the seed bank depleted [8], [11]. Bats and birds are airborne and thus the complexity of the forest, or lack thereof, has only a limited influence on their dispersive movements [12], [13]. In contrast, monkeys and other mammals, another key group responsible for seed dispersal, exhibit a limited contribution to recovery processes. This is because they are unwilling to enter areas of lowered complexity, where seeds are required, due to the elevated risk of predation in open habitats [14]. It appears that the scale of animal movements, and the factors that shape their spatial movement patterns, are key elements underpinning the recovery of degraded systems.

Mobile links may also be important determinants of ecosystem recovery on coral reefs [15], [16]. Herbivorous fish species are the main predators of macroalgae on coral reefs and are agents of system restoration in the early phases of macroalgal proliferation [17], [18], [19], [20]. Indeed, acute marcroalgal blooms (i.e. outbreaks) are becoming increasingly common on even seemingly healthy reefs [21], [22] and thus, the capacity of fish to mitigate these occurrences is critical. However, recent evidence suggests that when macroalgae replace corals as the dominant structure-forming benthic organisms, mobile herbivores are less willing to forage in these areas [15], [16], [20], [22]. It has been hypothesised that fishes avoid areas of high algal density because the complexity provided by the algae may conceal predators and thus, present an area of elevated predation risk [15]. This phenomenon may explain why high algal cover is correlated with a marked decline in fish biomass and diversity [16], [23].

Given the potential avoidance of degraded areas by mobile links, and the resultant lack of their respective ecosystem functions, it is important to understand how coral reef herbivores change their spatial patterns in response to outbreaks. It is known that the movement patterns of fish are strongly influenced by the presence of complexity and predation pressure (e.g. [24], [25], [26]), and the availability of food sources [27], [28]. However, it is not yet known if key fish taxa exhibit plasticity in their home range utilisation patterns, and are willing to shift their centres of activity to access temporally variable resources or, conversely, to reduce activity in areas of their habitat that become unfavourable.

The notion that the spatial tendencies [i.e. their movements and space utilisation] of taxa can have a marked impact on the recovery potential of ecosystems is troubling given the available evidence for coral reefs. The scales over which herbivorous fish taxa operate appear, with only rare exceptions, to be small, with site-attached behaviour being the most common (e.g., [26], [28], [29], [30]). Moreover, our understanding of the response of herbivores to the presence of algae on reefs is largely limited to small-scale simulated outbreaks [15], [31], [32]. Assessments of herbivore removal of macroalgae on healthy reefs are overwhelmingly based on assays comprised of a single thallus, or bunch of algae, and rarely exceed 1 m^2^ (e.g. [33], [34], but see [18], [20], [35]). These evaluations have identified several groups of herbivores with important functions (croppers, scrapers and excavators which graze algae, and browsers which consume adult macroalgae; [17]). Yet we know little of the behaviour and spatial scales over which these key taxa operate, especially in response to macroalgal outbreaks. Clearly larger-scale experimental manipulations are required to understand how the spatial tendencies of local herbivores may change in response to local algal outbreaks and to determine how reliant areas of reef are on the movement of fishes from afar.

The aim of this study, therefore, is to evaluate changes in the spatial tendencies of coral reef herbivores when exposed to an acute, simulated local algal outbreak. Specifically, we aim to determine if, and to what extent, the response of key fish taxa to algal outbreaks is a localized one (i.e., the response of herbivorous taxa will be limited to those taxa whose home range encompasses the outbreak) or a broader, community level response (i.e., the response occurs over broader areas of the reef and key species move in to feed on macroalgae).

## Materials and Methods

### Study locations

**Fig 1:**
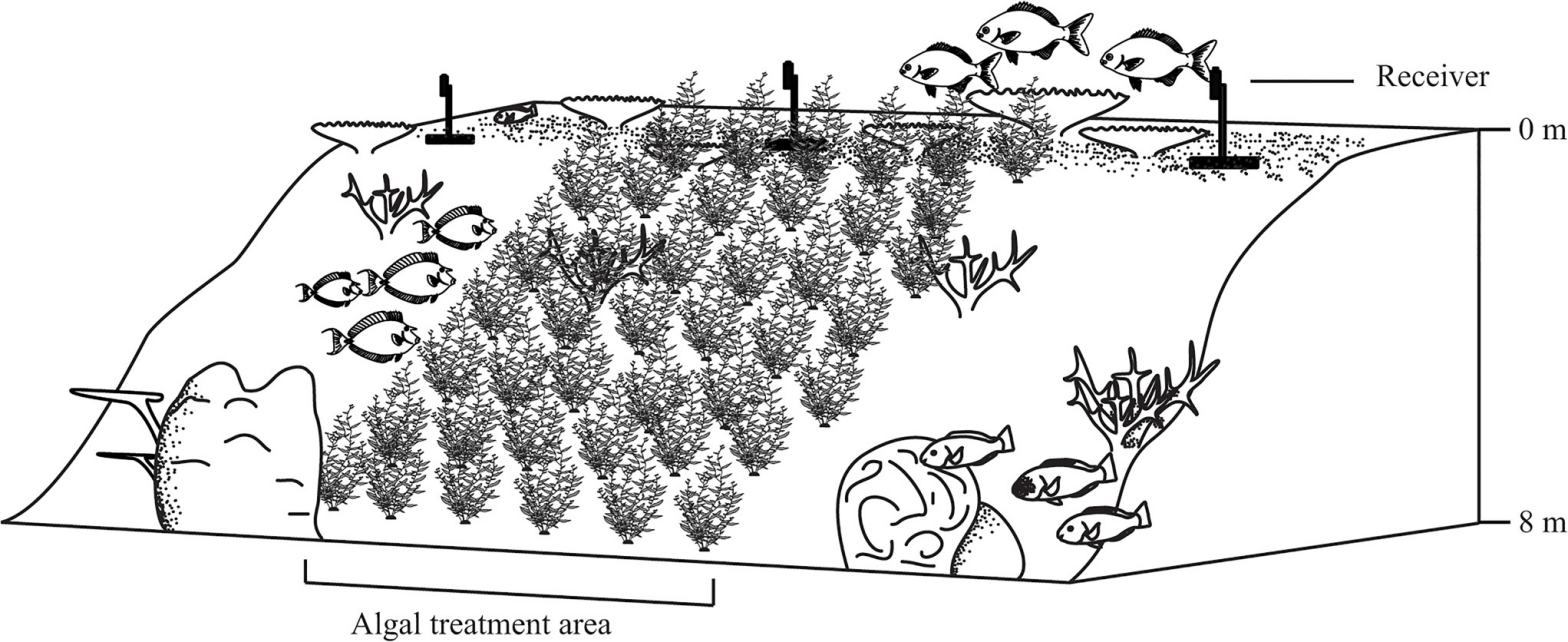
Simulated algal outbreak on a coral reef. Visual representation of the simulated macroalgal outbreak depicting initial macroalgal deployment density with acoustic receiver placement.

The study was conducted between April and November 2013, on reefs surrounding Lizard Island, a mid-shelf reef of the GBR (14°40’S 145°28’E). Two locations were selected to conduct the experiment, Mermaid Cove and Turtle Beach (please see supplementary information [SI] S1 Fig). Both locations are similar, on the leeward side of Lizard Island, with a distinct reef flat, crest, slope and base on sand at 6–8 m. Data for video analysis was collected largely on the reef crest (1–3 m) while algal deployment extended from the reef flat (0 m) to the reef slope (5 m) at both locations (Fig 1). At both study locations, macroalgae naturally occurs at a very low biomass [36] and therefore, the algae had to be collected from off-site.

All fieldwork was conducted under research permit number G11/34412.1, issued by the Great Barrier Reef Marine Park Authority.

### Simulated macro-algal outbreak

A degraded macroalgal-dominated reef was simulated by transferring *Sargassum* sp. (*cf*. *S*. *swartzii*) from the Turtle Island group, an inshore group of reefs 27 km southwest of Lizard Island (14°43’S, 145°12’E) to Lizard Island. *Sargassum* was chosen for the simulation as it has been shown to be the dominant successional macroalgal genus on the GBR [35]. *Sargassum* outbreaks have also been reported from the Indian Ocean [37]. *Sargassum* thalli of relatively uniform height (~50 cm) were removed from the benthos by gently prying the holdfast from the substratum. The algae were then transported to the Lizard Island Research Station (LIRS) where they were held in flow through tanks before being deployed. Algae were never held for more than a week and no algae were deployed which showed signs of degradation. Prior to being deployed, algal thalli were spun, weighted and attached together using twist ties to ensure that each deployed unit weighed approximately 0.5 kg. To fix the algae to the reef, 6 m long chains were placed in a grid configuration within a 50 m^2^ treatment area (algal plot), two days prior to algal-treatment video data collection. Following the pre-deployment recording period (see below for details) algae were attached to the chains using cable ties, which were attached to the holdfast of the thallus.

Between October and November, algae were fixed to the reef in a treatment plot (covering one of the observation/receiver sites) within each location, measuring approximately 50 m^2^ (Fig 1). The algal plot extended 5 m along the reef and 10 m down the reef gradient, encompassing the reef flat, crest and base. Initially, 200 thalli were deployed haphazardly within the algal plot at each location, resulting in an initial density of 4 thalli m^-2^ (approximately 2 kg m^-2^). However, supplemental algae were added to each site every second day to maintain densities of between 150–220 thalli per plot (density range; 3–4.4 thalli per m^2^; 1.5–2.2 kg m^-2^). At even the lowest algal density, sufficient *Sargassum* was present to ensure that the macroalgal composition of the benthos was numerically dominant to coral colony abundance. Algal plots were maintained in the plots for 14 days before being removed.

### Community response

To quantify the effect of an algal outbreak on the herbivore community, fishes were monitored using cameras and acoustic telemetry. For video recordings, four monitoring sites were chosen at each location. The monitoring sites roughly corresponded the placement of VR2W acoustic receivers (Vemco, Halifax) moorings deployed along the reef crest (details below). Cameras were placed to monitor a small-scale and large-scale response. The small-scale response was assessed with two recording sites, one within the algal treatment plot, and the other (used to quantify changes in the herbivore community in the immediate vicinity) was just outside the plot, 40 m away. Larger-scale effects on the herbivore community were assessed using two supplementary monitoring sites, roughly situated at acoustic receiver moorings 80 m along the reef on either side of the central monitoring sites (S1 Fig). This effectively provided one adjacent and two distant control sites.

Beginning in October 3013, a total of 30 video recording days were captured over a 34 day period. Monitoring was divided into three periods: ten pre-algal treatment days, ten days when the ‘algal-treatment’ was present and finally, ten post-disturbance days following algal removal. Video monitoring was suspended for two days prior to the algal treatment and two days following the algal treatment to allow the fish community to acclimate to the placement and removal (respectively) of the chains used to fix the algae to the reef. Within each of these monitoring periods, five days were randomly chosen for analysis. Video recordings were made using *GoPro* cameras, which were haphazardly deployed onto the reef within 10 m of each monitoring site (4 cameras per location), between 11:00 h and 16:00 h. After each camera had been deployed, a 1 m^2^ quadrat with markings at 5 cm intervals on all four sides was placed for 30 s on the benthos with the edge 50 cm from the lens of the camera. This ensured that the video sampling area was standardised and that size of fish that entered the sampling area (1 m^2^) could be estimated. Each camera was then left for a minimum of 3.5 h. Videos were examined on a computer with the sampling area marked on a plastic overlay on the screen. On the overlay, the 5 cm increments placed on the sampling quadrate were also marked to aid size estimations. Size estimates were validated by holding a model of a size unknown to the observer within the quadrate area. This was replicated 30 times. The estimated size of the model was then compared to its actual size. The absolute discrepancy between actual size and observer estimations was small (4.5 ± 0.49 cm; mean ± SE) and the relationship between estimated and actual size of the model yielded an r^2^ value of 0.92.

In each video the identity, size and number of each herbivorous fish species entering the videoed sampling area were recorded for the second 15 min period in each hour of recording, resulting in a total of 45 min per replicate. The videos therefore measured the prevalence of fishes in the area. It is the number of entrances of each species within a 1m^2^ area in 45 min. It is not an abundance measure but gives a relative indication of prevalence or presence of fishes in the area (expressed herein as fish density). Detailed notes were also recorded of the behaviour of different fish species within the videos.

### Response of residents

The response of focal individuals to the localized algal outbreak was also quantified using passive acoustic telemetry. In April, two arrays of 10 VR2W acoustic receivers were constructed at each location (total of 20 receivers). Prior to receiver placement, extensive range testing was conducted at both study locations and the working detection range (range at which 50% of known signal transmissions from a transmitter are detected; [38]) was found to be 40 m for the V7-4L acoustic transmitters (Vemco, Halifax) used in this study. Therefore, receivers were placed at increments of 40 m along the reef crest at each location. At each location, most of the reef crest was thus incorporated into the detection range of at least one receiver. Receivers were moored following the shallow water moorings described in Welsh et al. [38] and care was taken to ensure that the hydrophone of each VR2W unit was well above the algal canopy.

Once the array was in place, representative herbivore fish taxa were captured and tagged between April and September 2012. Prior to tagging, visual censuses at each location were used to estimate the average abundance of major herbivore species per location. Visual censuses were conducted 5 times over five months, prior to the study, to ensure patterns of fish abundance were consistent through time. At each location, a fish census consisted of nine 2 x 20 m transects along the reef crest. The start-point of each transects were haphazardly chosen within 20 m of each receiver mooring. Based on these data, representative individuals from herbivore functional groups that were likely to respond to algal presence (browsers, scrapers and croppers) were selected for tagging; these include *Naso unicornis* (browser), *Scarus schlegeli* (scraper), *Siganus vulpinus* and *S*. *corallinus* (grazer) [39], [40].

Fish for acoustic tagging were captured using barrier nets. Once captured, fish where transported to LIRS where they were held in flow-through tanks prior to surgery. To implant transmitters, fish were anaesthetized in a 70 L bath containing an MS-222 seawater solution (0.13 gL^-1^). Fish were considered anaesthetised when their righting reflex failed and gilling rate became reduced, which took approximately 5 mins. The fish were placed on a moist foam block, out of water, for the procedure. A small, 2 cm incision was then made mid-way between the upper margin of the pectoral fin insertion and the anus and a V7-4L acoustic transmitter inserted into the peritoneal cavity, below the swim bladder. The wound was closed with two dissolvable BIOSYN 3/0 sutures, tied using a Surgeon’s Knot. The surgical procedure took on average 180 (± 40 S.E.) seconds to complete once the fish had been removed from the anesthetic bath. Fish were released to the capture location following a 12–24 h recovery period. Based on visual observations of the tagged individuals, fish were fully recovered, showing no signs of the incision, one week after being released. All the methods utilized in the present study were approved by James Cook University Animal Ethics Committee (A1700). No tag-induced mortality was recorded.

### Data analysis

#### Herbivore community response

The taxa observed in video data recordings were classified into functional groups (grazers, scrapers, excavators and browsers; see S1 Table for species classification), to examine the response of functional groups to the presence of macroalgae.

The densities of species in the four functional groups were compared using a three-way MANOVA, with Location, Site and Treatment as independent fixed factors and Site nested within Location. For this analysis, the densities of individuals from each functional group were used as dependant variables. Within the MANOVA analysis, one-way ANOVA analyses were used to test the between-subject effects, identifying which functional groups differed significantly between Locations, Sites and algal treatments. Least significant differences analyses (LSD; based on mean comparisons; *t-test*) were then used to detect homogeneous subsets of functional group densities in the independent factors. To satisfy the assumptions of the MANOVA, density data were square-root transformed. Assessments of the square-root transformed data revealed that the MANOVA was a suitable analysis based on a non-significant Box’s test for equality of covariance matrices and a non-significant Levene’s test for homogeneity. Residual plots were also inspected to verify the suitability of the test.

To investigate species-level response of coral reef herbivores to macroalgae, two non-metric multidimensional scaling analyses (nMDS) were examined using video-based density data for each herbivore species (one included data on all herbivore species and the second considered only non-browsing species). In both cases, the significance of groupings identified in the nMDS were analysed using a one-way analysis of similarity (ANOSIM) preformed on an average distance matrix. Prior to nMDS and ANOSIM analyses, data were square-root transformed to improve normality in the data set and reduce the influence of high prevalence species on the analyses.

#### Resident response

To assess the response of tagged fish to the macroalgal disturbance, the detection data from 14 days prior to, during and following the algal deployment were used (excluding the two days during which algae was being deployed). For each fish an individual’s core receiver (with the majority of an individual’s detections during the 14 days prior the algal deployment) was identified. The change in occupancy at the core receiver was calculated for each tagged individual by subtracting the average number of detections per day prior to algal deployment from the average number of daily detections at the same receiver while the algae were in place. The resulting delta values were then compared to 0 (representing no change in occupancy) using four one-sample *t*-test (one separate analysis per species). Furthermore, the activity of tagged fish within the detection range of the receivers in the centre of each simulated algal outbreak (represented by number of detections per day) was calculated for each of the three temporal periods and compared using two repeated measures multivariate analysis of variance (RMANOVAs). Where necessary, the alpha values for the statistical tests were adjusted using Bonferroni correction.

## Results

### Herbivore community response

**Fig 2:**
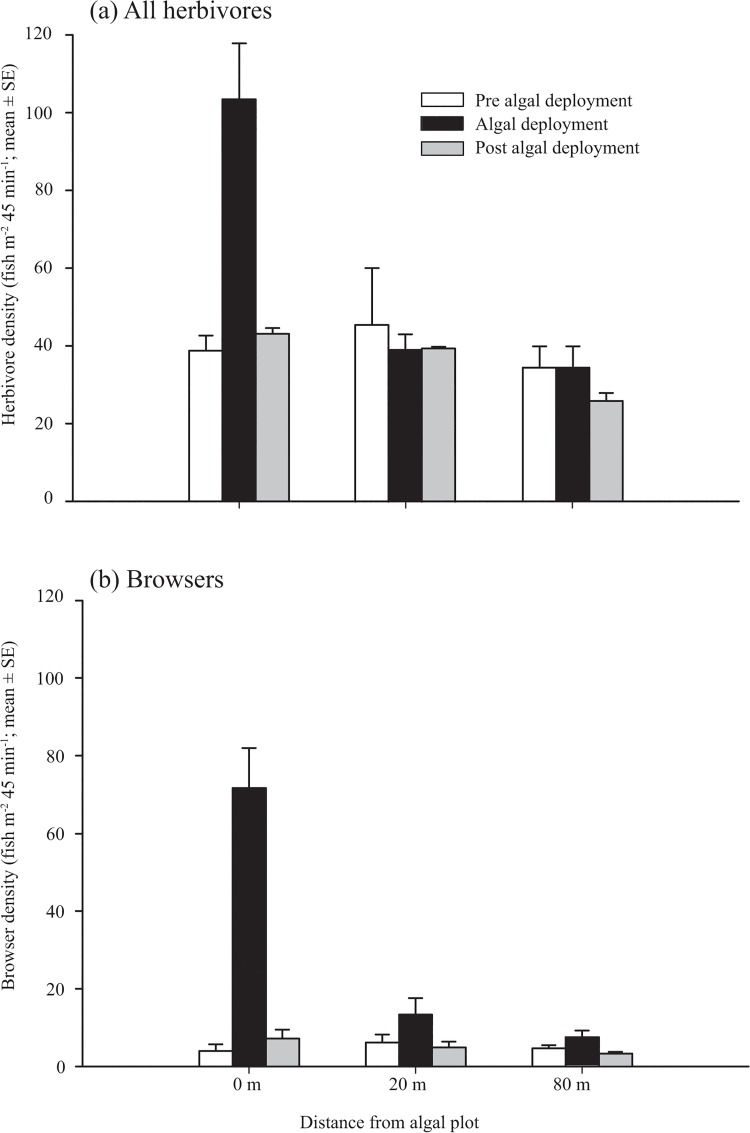
Densities of a) all herbivores and b) only browsing herbivores across algal treatments. The mean densities (fish m^2^ 45 min^-1^) of a) all herbivores and b) browsing herbivores before (white), during (black) and after (grey) a simulated macroalgal outbreak.

Video footage revealed no significant difference in the mean densities of species belonging to the four herbivore functional groups between the two locations (*Pillai’s trace*_4,99_ = 1.99, *P* > 0.05), nor was there an interaction effect between location and treatment (*Pillai’s trace*_8,200_ = 0.71, *P* > 0.05). However, during the macroalgal treatment, the mean densities (± SE) of herbivorous fishes at the treatment site increased from 38.8 ± 3.8 to 103.5 ± 14.3 (fish m^-2^ 45 min^-1^), a near 3 fold increase (Fig 2; S1 Table). Following the removal of the macroalgae, the density of fishes at the treatment site decreased to 43.1 ± 1.5, similar to initial density estimates (Fig 2). These patterns were not seen in any other monitoring location outside the algal treatment area, with fish densities remaining relatively constant throughout the study (Fig 2). The pronounced effect of algae on the densities of herbivores was supported by the MANOVA, which revealed a significant effect of algal treatment (before, during and after; *Pillai’s trace*_8,200_ = 10.54, *P* < 0.001), site (inside and outside treatment areas; *Pillai’s trace*_12,303_ = 3.02, *P* < 0.01) and an interaction effect (*Pillai’s trace*_24,408_ = 4.41, *P* < 0.001) (S2 Table), as sites were only significantly different from each other when algae were present (S1 Table). However, the effect was not even across the four functional groups. Univariate ANOVAs indicated that, of the four herbivore functional groups analysed, only browser densities changed significantly with algal treatments (*F*_2,120_ = 53.97, *P* < 0.001) and the interaction effect (*F*_6,120_ = 25.37, *P* < 0.001) (i.e., as increases in browser densities only occurred at sites where and when algae were present)(S3 and S4 Tables).

**Fig 3:**
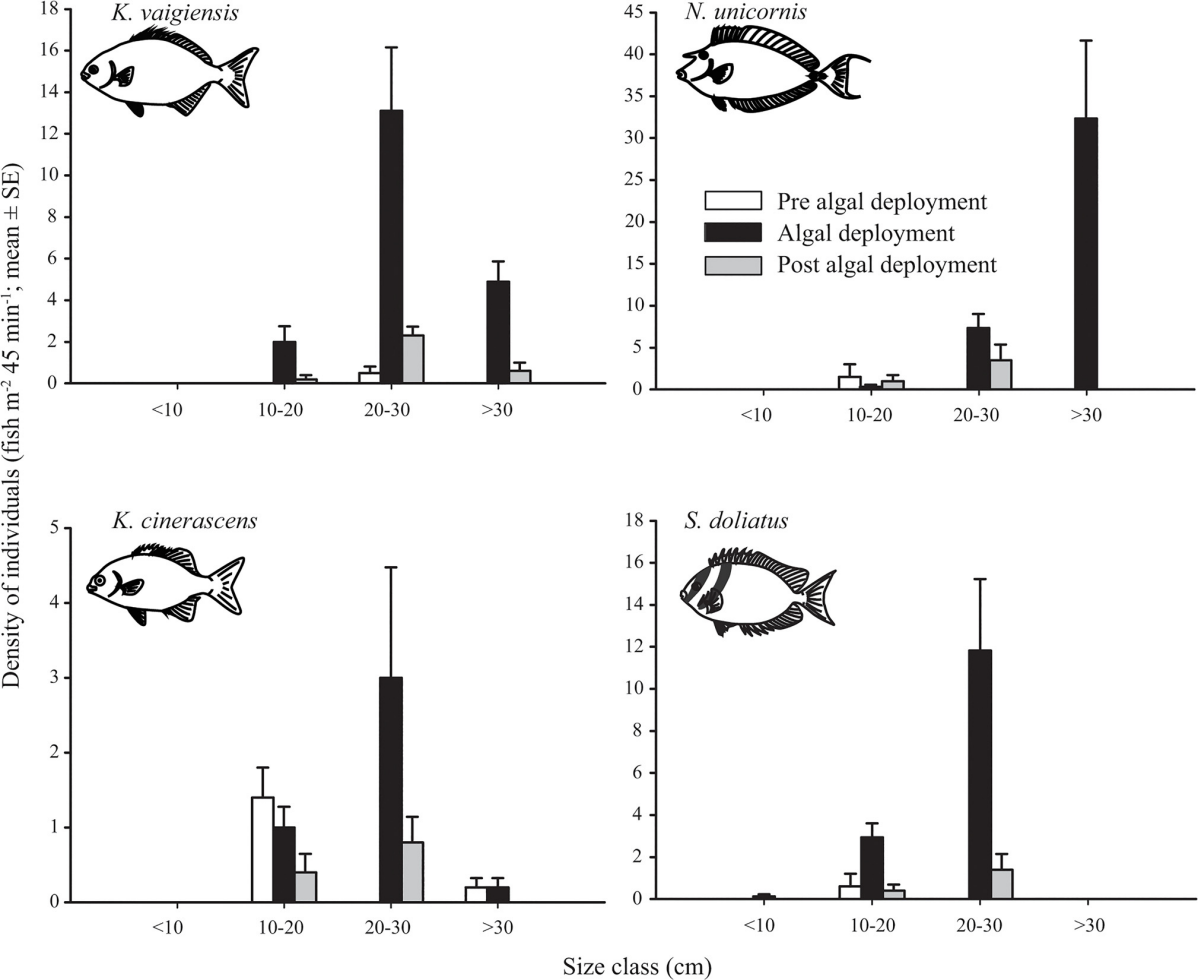
Size distribution of key browsers across algal treatments. Size frequency distribution of key browsing taxa; *Kyphosus vaigiensis*, *Naso unicornis*, *K*. *cinerascens* and *Siganus doliatus* before (white), during (black) and following (grey) a simulated phase shift to macroalgae.

In addition to changes in fish densities, the size structure of the browser population also changed dramatically when algae were placed on the reef. After deployment, the density of large (>30 cm) *N*. *unicornis* increased from 0 to an average of 33.2 ± 4.0 fish when algae were present (Fig 3). This pattern was also evident for *K*. *vaigiensis* and *S*. *doliatus*, for which no larger individuals (20–30 cm) were recorded prior to the algal treatment. However, once the algae were present their densities increased to 24.8 ± 3.1 and 26.0 ± 9.7, respectively, at the treatment site (Fig 3). A smaller, yet still notable increase in densities was recorded for *K*. *cinerascens* at sites of algal deployment (Fig 3).

**Fig 4:**
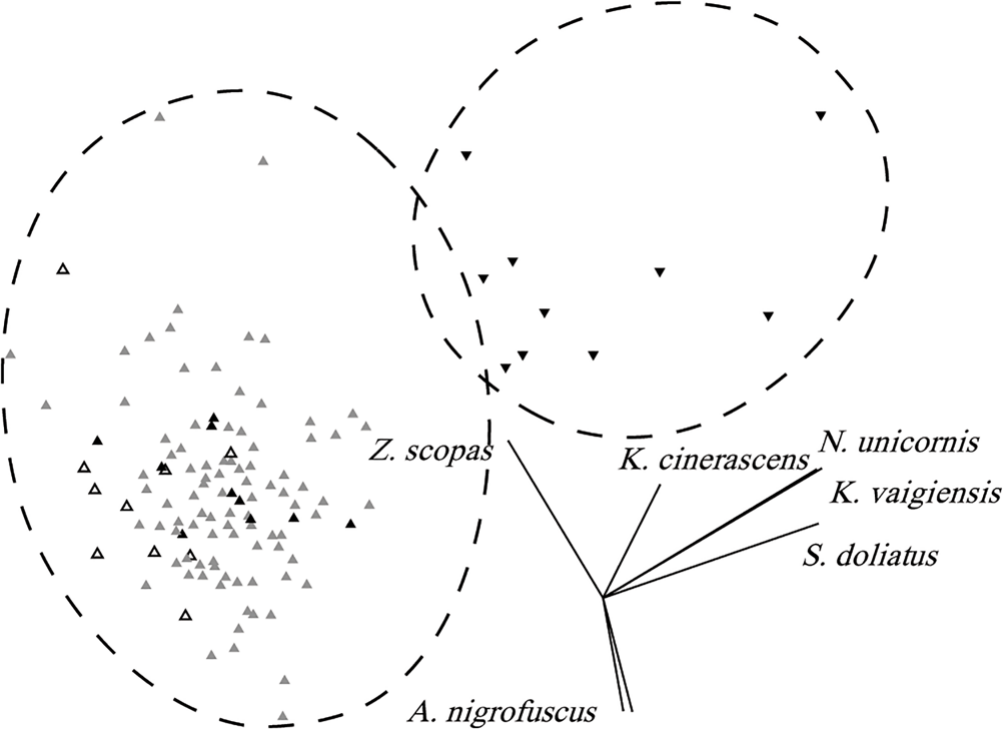
Differences in herbivore community composition by treatment site and algal presence. Non-metric multidimensional scaling (nMDS) analysis showing the relationship between 8 sites at three different treatment levels (pre, during and post algal treatment) based on the densities of herbivorous fish taxa. During algal deployment, sites are designated as either control or treatment. Ellipses represent significant groupings identified by ANOSIM. Vector lengths indicate the relative contribution of each species to the observed pattern. Grey triangles represent control sites while algae were present, inverted black triangles represent treatment sites while algae were present, unfilled triangles represent all sites prior to algal treatment and black triangles represent all sites post-algal treatment. 2D stress: 0.18.

The results from the species-level MDS analysis support the above observations with a strong separation of sites during algal deployment (Fig 4). ANOSIM detects a significant difference between sites in which algae were present and all others (global R = 0.937, significance level 0.1%; Fig 4). As expected, the separation of these sites was largely driven by the presence of browsing species, namely *N*. *unicornis*, *K*. *vaigiensis*, *S*. *doliatus*, and to a lesser extent, *K*. *cinerascens* (Fig 4). When all browsers were removed from the data set, no significant groupings were detected (global R = -0.125, significance level 89.2%)(S2 Fig).

During the period over which the algae were deployed, approximately 50 new thalli were added to each site every second day to replace lost algal thalli. When removed, the holdfasts of the algal thalli were still present and attached to the benthos; however, the standing stipe and blades were often removed. Observations of browser feeding behaviours within the algal plot indicated that, during the algal treatment, *N*. *unicornis* and *K*. *vaigiensis* fed on the algal in 100% and 83% (± 18 S.E.) of treatment days, respectively. To a lesser extent, *S*. *doliatus* and *K*. *cinerascens* were also regularly observed feeding on the algae on 66% (± 23) and 50% (± 24) of the treatment days, respectively.

### Resident response

**Fig 5:**
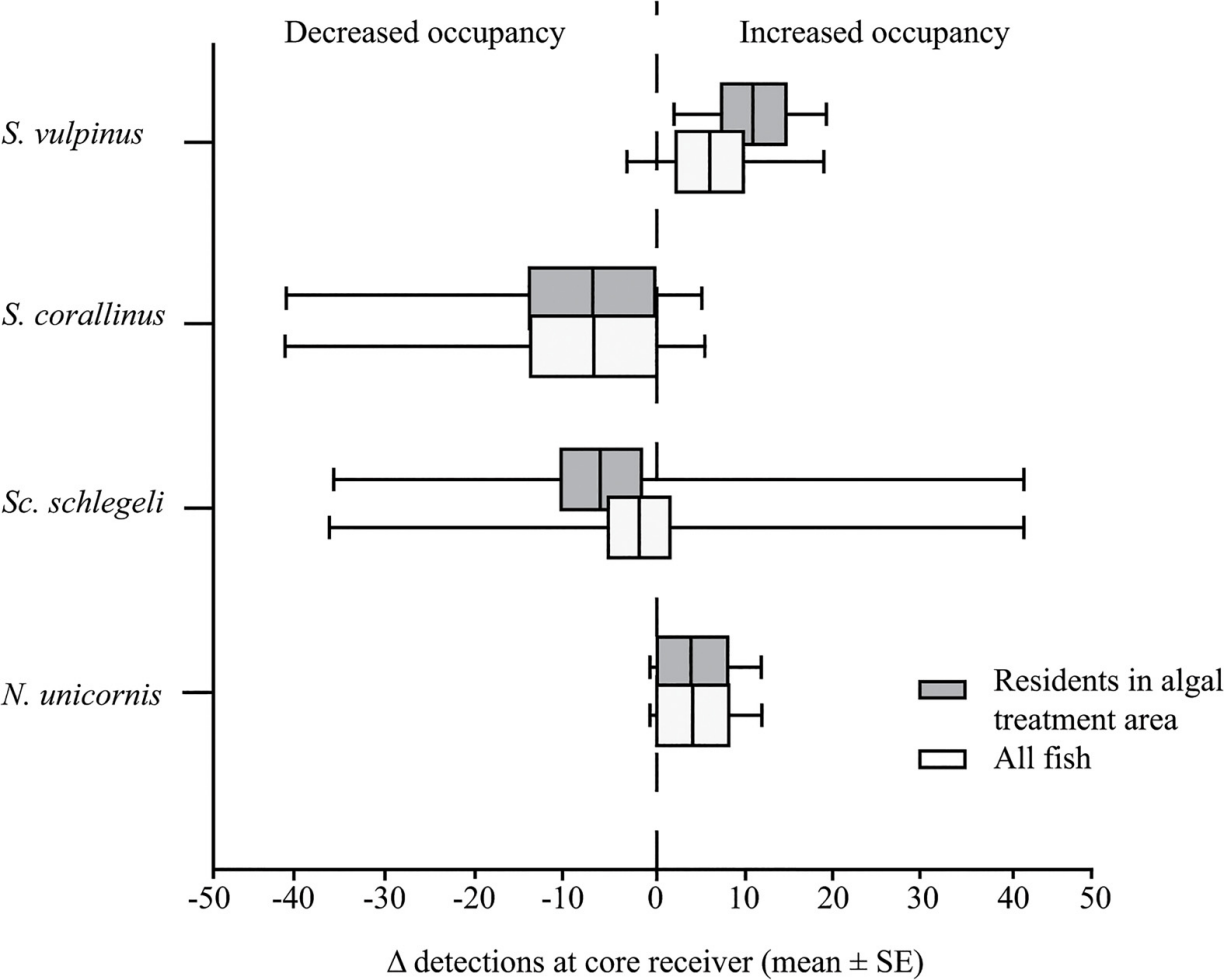
Change in fish residency with algal treatment. Change in individual detections rates (# detections day^-1^ during algal deployment—detections day^-1^ prior to deployment) for all *Signaus vulpinus* (*n* = 6), *S*. *corallinus* (*n* = 6), *Scarus schlegeli* (*n* = 17) and *Naso unicornis* (*n* = 3). Dark grey boxes represent fishes with core areas of detection was at the algal deployment site (*n* = 4, 6, 9 and 3 respectively). The box represents mean ± SE and whiskers represent minimum and maximum values. None of the species’ change in detection rates following algal deployment were significantly different from zero.

A total of 34 fish tagged with acoustic transmitters were monitored for the duration of the study. The number of each species tagged (*S*. *vulpinus n* = 6, *S*. *corallinus n* = 6, *Scarus schlegeli n* = 17 and *N*. *unicornis n* = 3) corresponded to the relative abundance of the taxa at each site and represented at least 40% of the estimated local population size (based on counts made during fish censuses; S5 and S6 Tables). Despite the marked changes in herbivore detections observed by videos, of the tagged fishes none significantly changed their patterns of occupancy during algal deployment, regardless of whether their core area of detection encompassed the simulated algal outbreak or not (Fig 5; S7 Table).

## Discussion

Our results demonstrate a rapid response in the herbivorous fish community to a simulated outbreak of macroalgae with changes in the browser community within days of algal introduction. This activity was highly focused, with no other sites on the same reef, even within 20 m, exhibiting similar increases in herbivore density. The observed increase in fish densities, however, did not arise from a response in resident taxa. Instead, it occurred as large mobile non-resident herbivores moved in to the site of algal deployment to feed on the available macroalgae. It appears that large mobile browsing herbivores can alter their behaviour to occupy areas exhibiting a localized algal outbreak. These observations suggest that coral reefs may rely on the activities of mobile links, i.e. large, mobile, non-resident individuals, to help mitigate the effects of localized algal outbreaks on coral reefs.

### A community response to algal outbreaks

The response of the fish community to the simulated macroalgal outbreak was marked. On average, the fish densities at the site of algal deployment increased by 267%. This response was surprising, as several studies have evaluated the effect of macroalgal growth on coral reefs and have overwhelmingly found a decline in fish abundance and/or diversity [16], [20,] [41], [42]. The cause for these declines has largely been attributed to a decline in reef complexity or suitable feeding surfaces following a phase shift over long temporal scales [16], [37], or the proliferation of undesirable complexity provided by macroalgae [15]. However, we found that for localized, acute increases in algae where the reef structure remains intact, the overall herbivore community shows no aversion to macroalgae; instead, large browsers are strongly attracted to the macroalgal site.

There are two possible explanations for these differences among studies evaluating acute algal deployments on coral reefs: 1) they may be a result of differences in algal identity, density, quality and/or extent, or 2) variation among studies in the composition of the herbivore assemblages. Previous work that has shown an aversion to large stands of macroalgae [22] may in part be due to algal densities that were higher than ours. This suggests that there is a threshold above which algal densities elicit a negative herbivore response and algal outbreak removal becomes difficult [15], [22]. Alternatively algae may differ in palatability where larger, older thalli are less desirable to herbivores [22], [43], [44], reducing the likelihood of herbivores responding to algal presence on reefs. Fish assemblage structure may also be important. The present study is the first on a mid-shelf reef, where the higher fish diversity provides a greater potential range of species to respond to algal deployments. Furthermore, macroalgae are rare on mid-shelf reefs relative to inner-shelf reefs [36]. Therefore, browsing herbivores may be more willing to feed on high density macroalgae on mid-shelf reefs, as the benefits of accessing the rare resource outweigh the elevated predation risks that may be associated with dense macroalgal fields [15].

The observed increase in fish densities was driven almost entirely by large macroalgal browsers. When algae were present, recorded densities of the browsing species, *N*. *unicornis*, were up to 10 times higher than in either pre- or post-monitoring periods. A similar, if less marked, increase in the densities of *K*. *vaigiensis* was also recorded. These species are known to feed primarily on macroalgae [16] and have been found to be uniquely capable of removing large quantities of macroalgae on the Great Barrier Reef [33], [45] and elsewhere in Australia [46]. This demonstrated a localized accumulation of functionally important taxa with the potential ability to reverse localized algal outbreaks [15]. Studies from the Seychelles [16] and the Red Sea [47] found a similar trend, in which degraded reefs with algal proliferation supported a higher abundance of browsing herbivorous taxa, as individuals presumably move in to feed on the algae. While movement of taxa for the purposes of resource exploitation is not a rare phenomenon in terrestrial or pelagic ecosystems (e.g. [48], [49], [50]), where large-scale movements are common, movements of this kind for the purpose of resource exploitation have only recently been recorded on coral reefs [49]. It appears that mobile fish taxa are capable of concentrating their residency and foraging activities at the site of macroalgal outbreaks in the same way terrestrial taxa change their behaviour to exploit specific resources. However, this raises the question of where did the individuals responsible for the increase in browser densities originate?

### The origin of responders

One of the most remarkable observations was that the increase in browsers in the algal deployment area was not a result of the movement of local residents. Of the three resident *N*. *unicornis* and 29 other herbivores tagged, none moved. This is evidenced by the consistent movement patterns recorded for all individuals monitored during the present study. The lack of a response from the resident community is also supported by the distinct shift in the size-frequency distribution of the browsing taxa. We recorded a clear peak in the number of large individuals (> 30 cm), which were, in the case of *N*. *unicornis*, not recorded on the reef prior to the algal deployment. A similar pattern was found for *K*. *vaigiensis*, *K*. *cinerascens* and *S*. *doliatus* in which the mean density of large individuals was greater than the combined values for these taxa across all sites prior to algal deployment. Indeed, *K*. *vaigiensis* was only recorded twice on the reef prior to the algal deployment in data from both video recordings and visual censuses. The dramatic increase in browser densities, and subsequent decline following algal removal, was recorded at both sites and arose from an influx of non-resident individuals that exhibited home ranges of a sufficient magnitude to allow them to encounter the algae.

Large-scale movements have been documented previously for *K*. *vaigiensis* on inshore reefs. Welsh and Bellwood [51] recorded movements in *K*. *vaigiensis* that were unprecedented for a coral reef herbivore and speculated that these movements may facilitate the detection of food sources with patchy distributions. This study supports this hypothesis and demonstrates the ability of these highly vagile taxa to change their movements in response to food availability. In contrast, the lack of movement in the smaller local fish is consistent with number recent studies, which report limited home ranges [25], [26], [28], [29]. This site-attached behaviour in reef fish highlights the importance of those species that do exhibit a spatial response.

It appears that upon detecting a food resource, large browsing herbivores can quickly alter their spatial range to exploit areas of high food availability. Previous research has suggested that *N*. *unicornis* is a highly site-attached species with a restricted home range [28], [52]. However, studies on this species’ movements are often restricted to small individuals although ontogenetic home range expansions have been documented [51]. Our observations are, again, consistent with these previous studies where smaller, resident *N*. *unicornis* do not move far, but larger individuals exhibit far greater mobility.

Once the macroalgae were removed, the density of browsing taxa returned to values almost indistinguishable from those before the simulated phase shift. This suggests that the browsers did not shift their home range to a new location in favour of a new resource but instead, temporarily changed their core areas of utilization within a much larger spatial range. This is significant, as it would suggest that the plasticity of movements within an individual’s home range might be key to preventing algal establishment and proliferation on coral reefs over large spatial scales, and may supplement the small-scale functional processes on reefs conferred by resident taxa.

### The importance of mobile links on coral reefs

The response of the mobile browsing herbivore community to the presence of macroalgae may be an important line of defence against reef phase- or regime shifts. Resident taxa may contribute to the control of algal outbreaks but may be unable to control them alone. It has been suggested that the growth rate of *Sargassum* may be too great for the algae to be removed entirely by resident taxa, and once established, fish may be unwilling to feed on the macroalgae [15]. Moreover, even on a small scale, the site-attached nature of numerous reef taxa is such that, even if an outbreak occurs on their reef, they may never encounter it [52]. Large, mobile herbivores may act as key mobile links, safeguarding against algal outbreaks, providing a cross-scale application of functional processes, with sufficient plasticity in their movements to focus their ecosystem services where needed.

This ecosystem response, however, is reliant on a healthy herbivore community, with large browsing individuals remaining in the system. It is not just a particular species of functional significance or adult individuals that are needed but large, mobile, individuals. This is especially concerning given that these large, mobile taxa are highly coveted by fishermen and are among the first to be removed from a system when fishing intensity increases [33], [53], [54], [55]. Furthermore, most marine protected areas (MPAs) are small, especially in tropical developing nations, where reef fish are highly targeted as a primary food source [54]. Therefore, by virtue of their mobility, these highly important individuals and species are more likely to move out of MPA boundaries and be removed first from the system. Fish that act as mobile links, therefore, appear to be some of the most important yet most vulnerable organisms on coral reefs.

## Supporting Information

S1 Dataset Raw Data Folder. Data has been separated into three sub-folders; video data, visual transect data and individual fishes’ acoustic detection data.
(ZIP)

S1 Fig Map of study sites. Map of the study locations used in Mermaid Cove and Turtle Bay at Lizard Island with algal deployment sites highlighted. Sold dots along the reef crest represents a receiver deployment location and white dots represent a combined video and receiver deployment location.
(EPS)

S2 Fig Differences in herbivore community composition excluding browseres by treatment site and algal presence. Non-metric multidimensional scaling (nMDS) analysis of showing the relationship between 8 locations at three different treatment levels (pre, during and post algal deployment) based on the densities of herbivorous fish taxa excluding browsers. Ellipses represent significant groupings identified by ANOSIM. Biplots indicate the relative contribution of each species to the observed pattern. Vector lengths represent the contribution of the densities of each taxon to the first two nMDS dimensions and are proportional to the squared multiple correlation coefficient (R^2^). Grey triangles represent control sites while algae were present, inverted black triangles represent treatment sites while algae were present, unfilled triangles represent all sites prior to algal treatment and black triangles represent all sites post-algal treatment. 2D stress: 0.21.
(EPS)

S1 Table Change in fish density with simulated algal outbreak. Average density (fish m^-2^ 45 min^-1^) of taxa at algal deployment sites during pre, during and post algal treatments recorded during video recordings. Data were pooled across sites.
(DOCX)

S2 Table Change in functional group density with simulated algal outbreak. Results from three-way MANOVA comparing the density of herbivore functional groups across study locations, sites and treatments.
(DOCX)

S3 Table Response of each functional group to algal presence. Multiple comparisons of MANOVA results using Least Significant Difference analysis, using *t-tests*, to identify significant differences between treatment subsets using square-root transformed density data from each herbivore functional group. Bold values are significant.
(DOCX)

S4 Table Site-specific response of functional groups to algal treatment. Results of one-way ANOVAs to identify the herbivore functional groups that significantly differ within the factors included in the three-way MANOVA. Bold values are significant.
(DOCX)

S5 Table Herbivores assessed for spatial response. Number of each herbivorous taxa captured and successfully monitored and their estimated abundance at each study site.
(DOCX)

S6 Table Average fish abundance in Mermaid Cove and Turtle Bay. Fish abundances derived from underwater visual transects.
(DOCX)

S7 Table Response of individual herbivores to algal treatment. One sample t-test comparing the mean change in detections at individual’s core receiver after algae had been deployed to 0. Data were separated into individuals which were (a) residents at the site of the phase shift and (b) residents at other areas of the reef.
(DOCX)
