# Supplementary material for: Simulated Macro-Algal Outbreak Triggers a Large-Scale Response on Coral Reefs
Source: PLoS One. 2015 Jul 14;10(7):e0132895. doi: 10.1371/journal.pone.0132895 (PMC4501832; doi:10.1371/journal.pone.0132895)
Supplement: S1 Table — Average density (fish m-2 45 min-1) of taxa at algal deployment sites during pre, during and post algal treatments recorded during video recordings. Data were pooled across sites. (DOCX) [file pone.0132895.s004.docx]

| **Table S1. Change in fish density with simulated algal outbreak.** Average density (fish m^-2^ 45 min^-1^) of taxa at algal deployment sites during pre, during and post algal treatments recorded during video recordings. Data were pooled across sites. | | | |
| --- | --- | --- | --- |
| **Species** | **Average Density** | | |
|  | **Pre** | **Algae** | **Post** |
| ***Grazers*** | | | |
| *Acanthurus pyroferus* | 1 | 0 | 0 |
| *A. aurenticavus* | 0 | 0 | 0 |
| *A. blochii* | 11 | 2.3 | 1 |
| *A. grammoptilus* | 0 | 0 | 0 |
| *A. lineatus* | 0 | 0 | 0 |
| *A. nigricauda* | 0 | 1.5 | 0 |
| *A. nigrofuscus* | 5.7 | 5.5 | 4.8 |
| *A. olivaceus* | 2 | 1 | 1.5 |
| *A. thompsoni* | 0 | 0 | 0 |
| *Centropyge bicolor* | 4 | 0 | 0 |
| *C. vroliki* | 2 | 0 | 0 |
| *Ctenochaetus striatus* | 8.5 | 7.9 | 8.3 |
| *Pomacanthus semicirculatus* | 0 | 0 | 0 |
| *P. sextriatus* | 1 | 1 | 1.5 |
| *Siganus argenteus* | 0 | 0 | 1 |
| *S. corallinus* | 3.2 | 2.8 | 3.4 |
| *S. lineatus* | 0 | 0 | 0 |
| *S. puellus* | 1 | 1 | 1.5 |
| *S. punctatus* | 1 | 1.6 | 1.5 |
| *S. punctatissimus* | 1 | 0 | 1.3 |
| *S. vulpinus* | 0 | 0 | 1.6 |
| *Zebrasoma scopas* | 1.8 | 2.3 | 1.8 |
| *Z. veliferum* | 1.5 | 1 | 1 |
| ***Scrapers*** | | | |
| *Hipposcarus longiceps* | 1 | 0 | 1 |
| *Scarus spp.* | 2.5 | 0 | 0 |
| *S. quoyi* | 1 | 0 | 0 |
| *S. chameleon* | 2 | 1 | 1.2 |
| *S. dimidiatus* | 1 | 1 | 1.5 |
| *S. flavipectoralis* | 0 | 0 | 1 |
| *S. frenatus* | 1.1 | 1 | 1 |
| *S. ghobban* | 1 | 2 | 1 |
| *S. globiceps* | 1 | 0 | 0 |
| *S. niger* | 1.6 | 1.8 | 2 |
| *S. oviceps* | 1.5 | 1.6 | 1.4 |
| *S. psittacus* | 1.4 | 2.2 | 1.3 |
| *S. rivulatus* | 2.9 | 3.5 | 2.8 |
| *S. rubroviolaceus* | 1 | 0 | 1 |
| *S. schlegeli* | 2.7 | 2 | 2.3 |
| *S. spinus* | 1 | 0 | 0 |
| ***Excavators*** | | | |
| *Cetoscarus bicolor* | 4 | 0 | 0 |
| *Chlorurus bleekeri* | 3 | 0 | 1 |
| *C. microrhinos* | 1.3 | 1.5 | 2.5 |
| *C. sordidus* | 3.9 | 2.1 | 3.8 |
| ***Browsers*** |  |  |  |
| *Calotomus carolinus^*^* | 0 | 1.6 | 1 |
| *Kyphosus cinerascens^*^* | 1.4 | 4.2 | 1.5 |
| *K. vaigiensis* | 1.25 | 20 | 3 |
| *Naso lituratus* | 3 | 2 | 2.3 |
| *N. unicornis* | 3.8 | 29.8 | 2.1 |
| *S. doliatus^*^* | 2 | 16 | 3 |
| ^*^Despite these taxa often being regarded as grazers, we noted them feeding on, and removing significant quantities of macroalgae and were thus, for our purposes, counted as browsers. | | | |
