# Supplementary material for: Simulated Macro-Algal Outbreak Triggers a Large-Scale Response on Coral Reefs
Source: PLoS One. 2015 Jul 14;10(7):e0132895. doi: 10.1371/journal.pone.0132895 (PMC4501832; doi:10.1371/journal.pone.0132895)
Supplement: S2 Table — Results from three-way MANOVA comparing the density of herbivore functional groups across study locations, sites and treatments. (DOCX) [file pone.0132895.s005.docx]

| **Table S2.** **Change in functional group density with simulated algal outbreak.** Results from three-way MANOVA comparing the density of herbivore functional groups across study locations, sites and treatments. | | | | | |
| --- | --- | --- | --- | --- | --- |
| **Source of variation** | **Test statistic (*Pillai’s trace*)** | ***F*** | **Hypothesis *df*** | **Error *df*** | ***P*** |
| Location (L) | 0.74 | 1.99 | 4 | 99 | 0.102 |
| Site_(Location)_ (S) | 0.320 | 3.018 | 12 | 303 | 0.001 |
| Treatment (T) | 0.593 | 10.537 | 8 | 200 | <0.001 |
| Interaction (S.T) | 0.824 | 4.412 | 24 | 408 | <0.001 |
| Interaction (L.T) | 0.55 | 0.705 | 8 | 2000 | 0.687 |
