# Supplementary material for: Simulated Macro-Algal Outbreak Triggers a Large-Scale Response on Coral Reefs
Source: PLoS One. 2015 Jul 14;10(7):e0132895. doi: 10.1371/journal.pone.0132895 (PMC4501832; doi:10.1371/journal.pone.0132895)
Supplement: S3 Table — Multiple comparisons of MANOVA results using Least Significant Difference analysis, using t-tests, to identify significant differences between treatment subsets using square-root transformed density data from each herbivore functional group. Bold values are significant. (DOCX) [file pone.0132895.s006.docx]

| **Table S3. Response of each functional group to algal presence.** Multiple comparisons of MANOVA results using Least Significant Difference analysis, using *t-tests*, to identify significant differences between treatment subsets using square-root transformed density data from each herbivore functional group. Bold values are significant. | | | | | |
| --- | --- | --- | --- | --- | --- |
| Dependent Variable | (I) Treatment | (J) Treatment | Mean Difference (I-J) | Std. Error | *P* |
|  |  |  |  |  |  |
| Grazers | Algal treatment | Post algal treatment | -0.0714 | 0.19293 | 0.712 |
|  |  | Pre algal treatment | -0.2547 | 0.19293 | 0.190 |
|  | Post algal treatment | Algal treatment | 0.0714 | 0.19293 | 0.712 |
|  |  | Pre algal treatment | -0.1833 | 0.19293 | 0.345 |
|  | Pre algal treatment | Algal treatment | 0.2547 | 0.19293 | 0.190 |
|  |  | Post algal treatment | 0.1833 | 0.19293 | 0.345 |
| Scrapers | Algal treatment | Post algal treatment | 0.0797 | 0.21140 | 0.707 |
|  |  | Pre algal treatment | -0.2810 | 0.21140 | 0.187 |
|  | Post algal treatment | Algal treatment | -0.0797 | 0.21140 | 0.707 |
|  |  | Pre algal treatment | -0.3607 | 0.21140 | 0.091 |
|  | Pre algal treatment | Algal treatment | 0.2810 | 0.21140 | 0.187 |
|  |  | Post algal treatment | 0.3607 | 0.21140 | 0.091 |
| Excavators | Algal treatment | Post algal treatment | -0.0782 | 0.21832 | 0.721 |
|  |  | Pre algal treatment | -0.2564 | 0.21832 | 0.243 |
|  | Post algal treatment | Algal treatment | 0.0782 | 0.21832 | 0.721 |
|  |  | Pre algal treatment | -0.1783 | 0.21832 | 0.416 |
|  | Pre algal treatment | Algal treatment | 0.2564 | 0.21832 | 0.243 |
|  |  | Post algal treatment | 0.1783 | 0.21832 | 0.416 |
| Browsers | Algal treatment | **Post algal treatment** | **1.8772^*^** | **0.21428** | **0.000** |
|  |  | **Pre algal treatment** | **2.0287^*^** | **0.21428** | **0.000** |
|  | Post algal treatment | **Algal treatment** | **-1.8772^*^** | **0.21428** | **0.000** |
|  |  | Pre algal treatment | 0.1516 | 0.21428 | 0.481 |
|  | Pre algal treatment | **Algal treatment** | **-2.0287^*^** | **0.21428** | **0.000** |
|  |  | Post algal treatment | -0.1516 | 0.21428 | 0.481 |
