# Supplementary material for: Simulated Macro-Algal Outbreak Triggers a Large-Scale Response on Coral Reefs
Source: PLoS One. 2015 Jul 14;10(7):e0132895. doi: 10.1371/journal.pone.0132895 (PMC4501832; doi:10.1371/journal.pone.0132895)
Supplement: S4 Table — Results of one-way ANOVAs to identify the herbivore functional groups that significantly differ within the factors included in the three-way MANOVA. Bold values are significant. (DOCX) [file pone.0132895.s007.docx]

| **Table S4. Site-specific response of functional groups to algal treatment.** Results of one-way ANOVAs to identify the herbivore functional groups that significantly differ within the factors included in the three-way MANOVA. Bold values are significant. | | | | | | |
| --- | --- | --- | --- | --- | --- | --- |
| Source | Dependent Variable | Type III Sum of Squares | *df* | *MS* | *F* | *P* |
| Location | Grazers | 1.066 | 1 | 1.066 | 1.423 | .236 |
|  | Scrapers | .276 | 1 | .276 | .283 | .596 |
|  | Excavators | .657 | 1 | .657 | .686 | .410 |
|  | Browsers | 2.351 | 1 | 2.351 | 2.483 | .118 |
| Treatment | Grazers | 1.381 | 2 | .690 | .922 | .401 |
|  | Scrapers | 2.873 | 2 | 1.436 | 1.475 | .234 |
|  | Excavators | 1.382 | 2 | .691 | .721 | .489 |
|  | **Browsers** | **102.167** | **2** | **51.084** | **53.966** | **.000** |
| Site_(Location)_ | Grazers | 5.614 | 3 | 1.871 | 2.498 | .064 |
|  | Scrapers | 5.285 | 3 | 1.762 | 1.810 | .150 |
|  | Excavators | 6.676 | 3 | 2.225 | 2.322 | .080 |
|  | Browsers | 9.698 | 3 | 3.233 | 3.415 | .020 |
| Location * Treatment | Grazers | 1.596 | 2 | .798 | 1.066 | .348 |
|  | Scrapers | .977 | 2 | .488 | .502 | .607 |
|  | Excavators | 1.080 | 2 | .540 | .563 | .571 |
|  | Browsers | 3.279 | 2 | 1.639 | 1.732 | .182 |
| Site_(Location)_ * Treatment | Grazers | 6.697 | 6 | 1.116 | 1.490 | .189 |
|  | Scrapers | 10.947 | 6 | 1.824 | 1.874 | .092 |
|  | Excavators | 6.659 | 6 | 1.110 | 1.158 | .335 |
|  | **Browsers** | **144.092** | **6** | **24.015** | **25.370** | **.000** |
| Error | Grazers | 76.401 | 102 | .749 |  | |
|  | Scrapers | 99.299 | 102 | .974 |  | |
|  | Excavators | 97.770 | 102 | .959 |  | |
|  | Browsers | 96.552 | 102 | .947 |  | |
| Total | Grazers | 1690.000 | 120 |  |  | |
|  | Scrapers | 1118.000 | 120 |  |  |  |
|  | Excavators | 404.000 | 120 |  |  |  |
|  | Browsers | 1704.000 | 120 |  |  |  |
