# Supplementary material for: Simulated Macro-Algal Outbreak Triggers a Large-Scale Response on Coral Reefs
Source: PLoS One. 2015 Jul 14;10(7):e0132895. doi: 10.1371/journal.pone.0132895 (PMC4501832; doi:10.1371/journal.pone.0132895)
Supplement: S5 Table — Number of each herbivorous taxa captured and successfully monitored and their estimated abundance at each study site. (DOCX) [file pone.0132895.s008.docx]

| **Table S5.** **Herbivores assessed for spatial response.** Number of each herbivorous taxa captured and successfully monitored and their estimated abundance at each study site. | | | |
| --- | --- | --- | --- |
| **Species** | **Site** | **Number tagged (average size; range [cm])** | **Mean abundance ± SE (max) within 40 m^2^ based on data from visual census** |
| *S. vulpinus* | Mermaid | 4 (22; 21.5 – 22.5) | 1 ± 1.0 (4) |
| *S. vulpinus* | Turtle | 2 (22.8; 22.5 – 23) | 0.25 ± 0.25 (2) |
| *S. corallinus* | Mermaid | 4 (20.3; 18.5 – 22) | 5 ± 2.6 (10) |
| *S. corallinus* | Turtle | 2 (22.3; 22 – 22.5) | 3.75 ± 2.5 (4) |
| *Sc. schlegeli* | Mermaid | 8 (24.4; 17.5 – 29.5) | 8.8 ± 1.8 (13) |
| *Sc. schlegeli* | Turtle | 9 (25.9; 19.5 –31) | 15 ± 1.7 (20) |
| *N. unicornis* | Mermaid | 2 (24.8; 20 – 28) | 3 ± 0.9 (5) |
| *N. unicornis* | Turtle | 1 (22.9; no range) | 1.5 ± 0.3 (2) |
