# Supplementary material for: Simulated Macro-Algal Outbreak Triggers a Large-Scale Response on Coral Reefs
Source: PLoS One. 2015 Jul 14;10(7):e0132895. doi: 10.1371/journal.pone.0132895 (PMC4501832; doi:10.1371/journal.pone.0132895)
Supplement: S6 Table — Fish abundances derived from underwater visual transects. (DOCX) [file pone.0132895.s009.docx]

| **Table S6. Average fish abundance in Mermaid Cove and Turtle Bay.** Fish abundances derived from underwater visual transects. | | |
| --- | --- | --- |
|  | **Average fish abundance ± SE within 40 m^2^** | |
| **Species** | **Mermaid** | **Turtle** |
| *Acanthurus blochii* | 1 ± 1 | 1 ± 1 |
| *Acanthurus lineatus* | 19.25 ± 4.71 | 5 ± 2.68 |
| *Acanthurus nigrocauda* | 2 ± 0.91 | 0.25 ± 0.25 |
| *Acanthurus nigrofuscus* | 17 ± 8.37 | 0.25 ± 0.25 |
| *Acanthurus olivaceus* | 1 ± 0.58 | 1.25 ± 0.48 |
| *Acanthurus pyroferus* | 0.25 ± 0.25 | 18.25 ± 5.12 |
| *Calotomus carolinus* | 0.25 ± 0.25 | 3.75 ± 1.89 |
| *Cetoscarus bicolor* | 1.25 ± 0.63 | 1 ± 0.41 |
| *Chlorurus bleekeri* | 0.25 ± 0.25 | 1.5 ± 0.29 |
| *Chlorurus microrhinos* | 1 ± 0 | 9.75 ± 3.40 |
| *Chlorurus sordidus* | 10.5 ± 2.22 | 24.25 ± 3.09 |
| *Ctenochaetus binotatus* | 0.25 ± 0.25 | 0.25 ± 0.25 |
| *Ctenochaetus striatus* | 61.75 ± 8.21 | 3 ± 1.08 |
| *Kyphosus vaigiensis* | 5.75 ± 2.02 | 4.5 ± 1.32 |
| *Naso brachycentron* | 0.25 ± 0.25 | 4 ± 4 |
| *Naso brevirostris* | 3.5 ± 2.36 | 5.25 ± 4.03 |
| *Naso lituratus* | 12 ± 7.31 | 1.5 ± 0.29 |
| *Naso unicornis* | 3 ± 0.91 | 1.5 ± 1.5 |
| *Pomacanthus sextriatus* | 2 ± 0.91 | 2.5 ± 1.19 |
| *Scarus dimidiatus* | 2.25 ± 0.94 | 1 ± 0.41 |
| *Scarus flavipectoralis* | 1 ± 0.41 | 0.75 ± 0.48 |
| *Scarus frenatus* | 4 ± 1.77 | 0.75 ± 0.48 |
| *Scarus ghobban* | 0.25 ± 0.25 | 1.25 ± 0.48 |
| *Scarus globiceps* | 0.75 ± 0.48 | 1.5 ± 0.96 |
| *Scarus niger* | 4.75 ± 1.25 | 0.25 ± 0.25 |
| *Scarus oviceps* | 3 ± 0.82 | 3.75 ± 1.38 |
| *Scarus quoiy* | 0.25 ± 0.25 | 1 ± 0.71 |
| *Scarus rivulatus* | 6.25 ± 2.50 | 0.5 ± 0.29 |
| *Scarus rubroviolaceus* | 1.25 ± 0.48 | 24 ± 4.60 |
| *Scarus schlegeli* | 8.75 ± 1.75 | 0.25 ± 0.25 |
| *Siganus argentious* | 0.75 ± 0.75 | 15 ± 1.73 |
| *Siganus corallinus* | 5 ± 2.61 | 0.25 ± 0.25 |
| *Siganus doliatus* | 8.5 ± 1.5 | 3.75 ± 0.25 |
| *Siganus puellus* | 1.5 ± 0.96 | 25.5 ± 3.97 |
| *Siganus punctatus* | 0.5 ± 0.29 | 0.25 ± 0.25 |
| *Siganus vulpinus* | 1 ± 1 | 0.25 ± 0.25 |
| *Zebrasoma scopas* | 7.75 ± 2.50 | 4.75 ± 2.32 |
| *Zebrasoma veliferum* | 1 ± 0.41 | 0.5 ± 0.5 |
