# Supplementary material for: Simulated Macro-Algal Outbreak Triggers a Large-Scale Response on Coral Reefs
Source: PLoS One. 2015 Jul 14;10(7):e0132895. doi: 10.1371/journal.pone.0132895 (PMC4501832; doi:10.1371/journal.pone.0132895)
Supplement: S7 Table — One sample t-test comparing the mean change in detections at individual’s core receiver after algae had been deployed to 0. Data were separated into individuals which were (a) residents at the site of the phase shift and (b) residents at other areas of the reef. (DOCX) [file pone.0132895.s010.docx]

| **Table S7.** **Response of individual herbivores to algal treatment.**  One sample t-test comparing the mean change in detections at individual’s core receiver after algae had been deployed to 0. Data were separated into individuals which were (a) residents at the site of the phase shift and (b) residents at other areas of the reef. | | | |
| --- | --- | --- | --- |
| **Species** | ***t*** | ***df*** | ***P*** |
| (a) | | | |
| *S. vulpinus* | 1.528 | 3 | 0.224 |
| *S. corallinus* | -1.014 | 5 | 0.357 |
| *Sc. schlegeli* | -1.564 | 8 | 0.156 |
| *N. unicornis* | 0.989 | 2 | 0.427 |
| (b) | | | |
| *Sc. schlegeli* | -1.314 | 10 | 0.218 |
